# Modeling mutant phenotypes and oscillatory dynamics in the *Saccharomyces cerevisiae* cAMP-PKA pathway

**DOI:** 10.1186/1752-0509-7-40

**Published:** 2013-05-17

**Authors:** Kevin Gonzales, Ömür Kayıkçı, David G Schaeffer, Paul M Magwene

**Affiliations:** 1 Department of Mathematics, Duke University, Durham, NC 27708, USA; 2 Department of Biology, Duke University, Durham, NC 27708, USA; 3 IGSP Center for Systems Biology, Duke University, Durham, NC 27708, USA

**Keywords:** Yeast, Signal transduction, Second messenger, Genetic variation

## Abstract

Background The cyclic AMP-Protein Kinase A (cAMP-PKA) pathway is an evolutionarily conserved signal transduction mechanism that regulates cellular growth and differentiation in animals and fungi. We present a mathematical model that recapitulates the short-term and long-term dynamics of this pathway in the budding yeast, *Saccharomyces cerevisiae*. Our model is aimed at recapitulating the dynamics of cAMP signaling for wild-type cells as well as single (*pde1Δ* and *pde2Δ*) and double (*pde1Δpde2Δ*) phosphodiesterase mutants.

Results Our model focuses on PKA-mediated negative feedback on the activity of phosphodiesterases and the Ras branch of the cAMP-PKA pathway. We show that both of these types of negative feedback are required to reproduce the wild-type signaling behavior that occurs on both short and long time scales, as well as the the observed responses of phosphodiesterase mutants. A novel feature of our model is that, for a wide range of parameters, it predicts that intracellular cAMP concentrations should exhibit decaying oscillatory dynamics in their approach to steady state following glucose stimulation. Experimental measurements of cAMP levels in two genetic backgrounds of *S. cerevisiae* confirmed the presence of decaying cAMP oscillations as predicted by the model.

Conclusions Our model of the cAMP-PKA pathway provides new insights into how yeast respond to alterations in their nutrient environment. Because the model has both predictive and explanatory power it will serve as a foundation for future mathematical and experimental studies of this important signaling network.

## Background

**Figure 1:**
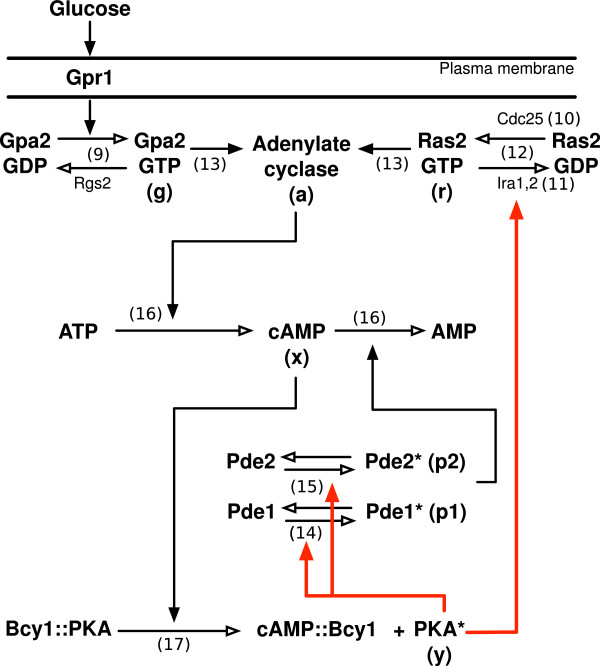
**Key interactions of the cAMP-PKA pathway in yeast.** See the main text and Additional file 1: Supplementary Model for descriptions of each reaction. Arabic numerals refer to equations in the Additional file 1: Supplementary Model that describe each reaction. The key interactions in the model presented here are depicted with red lines.

In eukaryotic cells, the cyclic adenosine monophosphate (cAMP) – Protein Kinase A (PKA) pathway plays a central role in mediating diverse biological responses such as growth, development, and cell differentiation [1,2]. Stimuli such as hormones, neurotransmitters, nutrients, and physiological stress agents trigger signaling cascades that collectively mount a response through cAMP-mediated PKA signaling [3-9]. In the budding yeast, *Saccharomyces cerevisiae*, activation of PKA in response to essential nutrients and fermentable carbon sources is directed by intracellular levels of cAMP [10]. Synthesis of cAMP from ATP is catalyzed by the enzyme adenylate cyclase (Cyr1) and governed by two different G-protein systems (Figure 1) [10-12]. Ras2, a small GTP-binding protein, in its GTP-bound (active) state stimulates adenylate cyclase, causing a rapid increase in intracellular cAMP levels (Figure 1, top right) [11]. Ras2 activity is positively regulated by the guanine nucleotide exchange factors (GEFs) Cdc25 and Sdc25 as a function of intracellular glucose levels [13-20]. Conversely, GTPase activating proteins, Ira1 and Ira2, down regulate Ras2 activity by stimulating the hydrolysis of GTP [21,22]. In parallel to Ras, a second G-protein pathway, involving the proteins Gpr1, Gpa2, and Rgs2, responds to extracellular levels of glucose and increases adenylate cyclase activity (Figure 1, top left). Gpr1 is a membrane bound G-protein coupled receptor that activates the G *α* protein Gpa2 in response to extracellular glucose levels. The GTP bound form of Gpa2 in turn stimulates adenylate cyclase activity [12,20,23,24]. cAMP synthesis through this pathway is negatively controlled by Rgs2, the GTPase activating protein of Gpa2 [25,26]. An increase in cellular cAMP levels upon glucose induction activates cAMP-dependent PKA. PKA is a holoenzyme that includes both regulatory (Bcy1) and catalytic subunits (Tpk1, Tpk2 and Tpk3) [27,28]. cAMP binding to the regulatory subunits leads to the release of the catalytic PKA subunits ([29]; Figure 1, bottom) which are then free to interact with downstream targets such as metabolic enzymes, transcription factors and other kinases [30-33].

Downstream of the adenylate cyclase, cAMP levels are modified by the action of phosphodiesterases, enzymes that catalyze the conversion of cAMP to AMP, thus preventing cAMP accumulation in the cell [34,35]. In yeast there are two phosphodiesterases, the low-affinity phosphodiesterase Pde1 and the higher affinity Pde2. Pde2 is a class II phosphodiesterase and shares homology with other eukaryotic phosphodiesterases [36], while Pde1 homologues have been identified in only a small number of eukaryotes [35]. The high-affinity Pde2 is thought to play the key role in maintaining cAMP at steady state levels [37]. The role of Pde1 is less well understood, though genetic studies in both *S. cerevisiae* and the distantly related yeast *Candida albicans* suggests that Pde1 is a target of PKA, and that PKA-mediated feedback on Pde1 is an important component of maintaining tight regulation of cAMP levels [35,38].

In this study we present a mathematical model of the cAMP-PKA pathway in *Saccharomyces cerevisiae*. The primary goal of this model is to explain the observed response of wild-type cells and PDE mutants (*pde1Δ*, *pde2Δ*, and *pde1Δpde2Δ*) to glucose stimulation. Wild-type cells exhibit a rapid, transient increase in cAMP levels followed by a quick return to a new steady state; this response typically occurs on a time scale of 60-90 seconds [35]. *pde2Δ* mutants have approximately wild-type dynamics, while *pde1Δ* mutants exhibit elevated peak cAMP levels and a slow return to a steady state that is higher than that of wild-type cells. Surprisingly, the double phosphodiesterase mutant, *pde1Δpde2Δ*, shows no response to glucose stimulus, and maintains cAMP concentrations at essentially a pre-stimulus steady state [35]. The question we posed was whether it was possible to mathematically reconcile all of these observed dynamics with currently known genetic and biochemical interactions. Our model focuses on PKA-mediated negative feedback interactions, and suggests that feedback on both phosphodiesterases and on the Ras branch of the pathway are important for reproducing the dynamics observed in wild-type cells and PDE mutants.

While a number of studies have proposed mathematical models for the cAMP-PKA network in yeast [39-41], none of these previous models is capable of recapitulating the observed dynamics of both wild-type cells and the double phosphodiesterase mutant (*pde1Δpde2Δ*). Our proposed model is thus unique in this aspect. Another notable feature of our model is that, for a wide range of parameters, it predicts the presence of decaying cAMP oscillations following glucose stimulus. Oscillatory behaviors were not an explicit goal of our initial modeling effort, and thus we treated this as a novel model prediction. We tested this prediction by measuring the cAMP response to glucose stimulus in two genetically diverse yeast strains (S288c and *Σ*1278b). Our experiments reveal variation in oscillatory behaviors between these genetic backgrounds, and we demonstrate that our model, in turn, is capable of reproducing the diversity of observed oscillatory patterns. Finally, our core model can also be easily extended to generate sustained cAMP oscillations that occur on longer time scales (tens of minutes; [42]). The results presented here thus demonstrate both the descriptive and predictive power of the model we have developed, and suggest that this model can serve as a basis for future experimental and modeling studies of this important eukaryotic signal transduction pathway.

## Summary of motivating experiments

There is a large body of experimental work that provides information on genetic and biochemical interactions relevant to the cAMP-PKA pathway. The strategy we adopted in this study was to use this body of work as a basis for constraining interactions in our model. In particular our model was developed to understand two specific experimental results: the short-term dynamics of cAMP following glucose stimulation reported by Ma et al. [35]; and the long-term dynamics of cAMP under various stress levels reported by Garmendia-Torres et al. [42].

### Short-term cAMP dynamics

**Figure 2:**
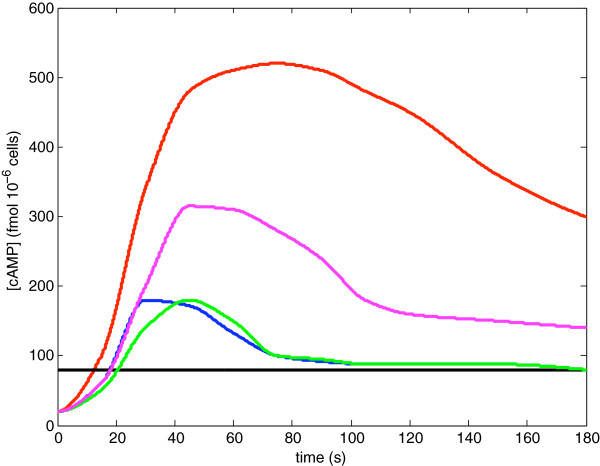
**cAMP dynamics following glucose stimulus as described by Ma et al. wild type (blue); *pde1Δ*(red), *pde2Δ*(green), *pde1***^**ala152**^**(pink),*pde1Δpde2Δ *(black).**

**Figure 3:**
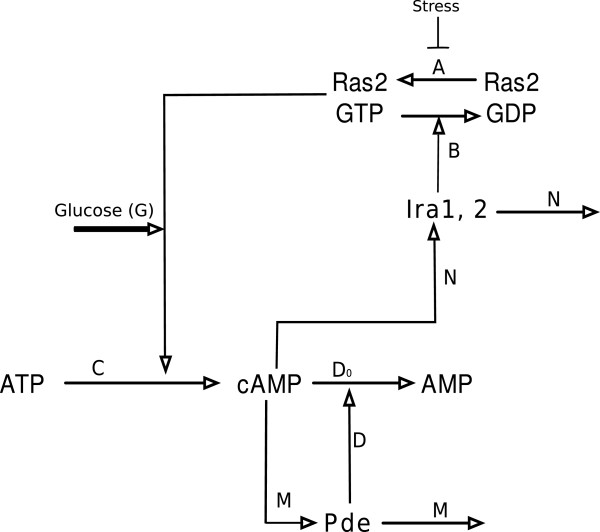
Key interactions of the cAMP-PKA pathway in yeast modeled by Equations (4a)–(4d).

Ma et al. [35] investigated the short-term behavior of cAMP signaling following glucose stimulation in cells that had previously been grown under carbon-source limitation. These data therefore represents the dynamics of cAMP signaling as cells transition from starved to fed states, on a time scale of seconds to minutes. Ma et al.’s analysis focused particularly on PKA-mediated negative feedback involving the phosphodiesterases Pde1 and Pde2. Figure 2 summarizes the five dynamic patterns we consider here, based on Ma et al.’s experiments involving wild-type cells and various phosphodiesterase mutants (compare to Ma et al. Figures 2a and 3a). These cases are: 

**Case 1:** Wild-type (*wt*; blue line) – the concentration of cAMP in *wt* cells rapidly increases following glucose stimulation. After reaching a peak level, the concentration of cAMP declines to a new steady state that is higher than its initial concentration. We refer to this as the wild-type transient response.

**Case 2:** Pde1 knockout (*pde1Δ*; red line) – the concentration of cAMP exhibits a much larger and longer transient response, about four times as large as the transient response exhibited by wild-type.

**Case 3:** Pde2 knockout (*pde2Δ*; green line) – the concentration of cAMP exhibits similar dynamics to wild-type.

**Case 4:** Pde1 phosphorylation mutant (*pde1*^*ala*152^; pink line) – the cAMP transient response is about twice as large as that exhibited by wild-type.

**Case 5:** Double PDE knockout (*pde1Δpde2Δ*; black line) – glucose stimulation has no effect on the concentration of cAMP present in the cytosol. The initial value in this case is much larger then the wild-type initial value.

These results are surprising in two ways: 1) the dynamics observed in the *pde2Δ* mutant are almost the same as those observed in wild-type cells, while the response in the *pde1Δ* mutant is drastically different, despite the fact that Pde2 is thought to be the higher affinity phosphodiesterase; and 2) in the double mutant (*pde1Δpde2Δ*) glucose stimulation has no effect on the concentration of cAMP, indicating a synergistic interaction between the PDE null-alleles.

### Long-term cAMP dynamics

Garmendia-Torres et al. [42] focused on the long-term behavior of cAMP signaling in yeast exposed to stress conditions. They predicted sustained oscillations, with a period of 5-10 minutes, in the concentration of cAMP at intermediate stress levels. This prediction was motivated by oscillations observed in the nucleocytoplasmic shuttling of the transcriptional activator Msn2. Msn2 is negatively regulated by the cAMP-PKA pathway through phosphorylation of Msn2 by PKA; this phosphorylation promotes export of Msn2 from the nucleus [43]. Garmendia-Torres et al. predicted that the nucleocytoplasmic shuttling of Msn2 that they observed was driven by oscillations in the concentration of cAMP, and they constructed a mathematical model to explore the proposed dynamics. In their model, high levels of stress drive the concentration of cAMP to a low steady state, and at low stress levels the cAMP levels go to a high steady state. Only under intermediate levels of stress did cAMP oscillations occur. This oscillatory behavior in their model was driven by PKA-mediated negative feedback on Ras ·GTP.

### Mechanisms of PKA-mediated negative feedback on cAMP signaling

Both sets of observations described above suggest that PKA plays an important role in attenuating cAMP signaling via negative feedback interactions. PKA-mediated negative feedback on cAMP signaling has been proposed to occur at two levels – via enhancement of phosphodiesterase activity and via attenuation of Ras signaling.

PKA interactions with Pde1 are supported by experiments by Ma et al. that showed that mutagenesis of a putative PKA phosphorylation site in Pde1 (*pde1*^*ala*152^) causes a dramatic increase in cAMP accumulation (Case 4, Figure 2). There is less direct evidence for PKA interactions with Pde2 but other class II phosphodiesterases are known to be targets of PKA [36]. Hu et al. [44] showed that hyperactive PKA activity leads to elevated Pde2 levels, and that this resulted from increased protein stability of Pde2.

The observation that cAMP signaling did not significantly change in the *pde2Δ* mutant might seem to suggest that Pde2 plays no role in regulating cAMP immediately following glucose stimulus. However, the cAMP transient in the *pde1Δ* mutant, and the synergistic effect observed in the *pde1Δpde2Δ* double mutant, argue for a role for Pde2, though with a slower activation rate as compared to Pde1. However, while we favor the hypothesis of PKA feedback on both PDEs, we can not rule out the possibility that the transient cAMP dynamics observed in the *pde1Δ* mutant might involve other feedback mechanisms.

With respect to Ras signaling, one or more elements of this pathway may be targets of PKA-mediated feedback. For example, Ira1 and Ira2 both have predicted PKA phosphorylation sites [12,45]. Similarly, PKA hyper-phosphorylates Cdc25, causing its dissociation from the adenylate cyclase/Ras2 complex. Cdc25 may also directly bind to the adenylate cyclase to facilitate membrane anchoring [46-48]. Re-localization of Cdc25 to the cytoplasm attenuates the glucose response of adenylate cyclase, inhibiting synthesis of cAMP [49,50]. Moreover, membrane dissociated Cdc25 can no longer activate Ras2, triggering an increase in Ras ·GDP levels [4]. Ma et al. suggested that the likely explanation for the pattern they observed in the *pde1Δpde2Δ* double mutant (no change in cAMP levels after glucose stimulation) resulted from constitutively high PKA feedback inhibition in the absence of the phosphodiesterases.

In summary, both studies described above suggest that PKA down regulates cAMP signaling by increasing the rate of PDE mediated decay and/or by decreasing the rate of cAMP production via interactions with the Ras effectors. Thus a key goal of our modeling effort was to explore PKA feedback on phosphodiesterase activity and the Ras pathway. In our model PKA interacts with both PDEs to increase their activity, though we assume PKA has a higher affinity for Pde1 than for Pde2. Similarly, we model negative feedback on Ras ·GTP through PKA phosphorylation of the Ras-GTPases Ira1 and Ira2. These interactions are highlighted by the red arrows in Figure 1.

## Model

To fully describe the key reactions of the cAMP-PKA pathway as depicted in Figure 1 requires a system of nine time-dependent differential equations to model the concentrations of: 1) Gpa2 ·GTP (active Gpa2); 2) Cdc25; 3) Ras ·GTP-ases Ira1 and Ira2; 4) Ras ·GTP (active Ras2); 5) adenylate cyclase activity; 6) activated Pde1; 7) activated Pde2; 8) cAMP; and 9) active PKA (free catalytic subunits). The full nine-dimensional system is described in detail in Additional file 1: Supplementary model (Equations (S1)-(S9)).

For the purposes of exposition, we first describe the motivation behind the equations that represent PKA mediated feeedback. We then describe a simplification of the model to a four-dimensional system that replicates the key dynamical behaviors of cAMP signaling as described above.

### Key reactions for PKA-mediated feedback

PKA feedback (red arrows in Figure 1) in our model occurs in three ways: (i) PKA phosphorylates Pde1, enhancing Pde1 breakdown of cAMP; (ii) PKA phosphorylates Pde2, enhancing Pde2 breakdown of cAMP; and (iii) PKA phosphorylates the Ras ·GTP-ases Ira1 and Ira2. In Cases (i) and (ii), PKA inhibits the concentration of cAMP by enhancing the breakdown of cAMP. In Case (iii) PKA inhibits the production of cAMP by inactivating Ras ·GTP.

We assume that there are much greater concentrations of Pde1, Pde2, Ira1 and Ira2 in the cytoplasm than of PKA. We also assume that Pde1, Pde2 and Ira1/2 compete with each other for activation by PKA. We further assume that Ira1 and Ira2 can be simultaneously activated by PKA, thus we treat them as a single variable, Ira. These assumptions are taken in order to understand the Ma et al. cases. The three competitive reactions are specified as follows:

$$\begin{array}{l} \left[\text{Pde}1]+[\text{PKA}]\overset{k_{1}}{\underset{k_{-1}}{⇌}}[\text{Pde}1::\text{PKA}]\overset{R_{p_{1}}}{\to }[\text{Pde}1^{*}]+[\text{PKA}\right]\\ \left[\text{Pde}2]+[\text{PKA}]\overset{k_{2}}{\underset{k_{-2}}{⇌}}[\text{Pde}2::\text{PKA}]\overset{R_{p_{2}}}{\to }[\text{Pde}2^{*}]+[\text{PKA}\right]\\ \;[\text{Ira}]+[\text{PKA}]\overset{k_{z}}{\underset{k_{-z}}{⇌}}[\text{Ira}::\text{PKA}]\overset{R_{z}}{\to }[{\text{Ira}}^{*}]+[\text{PKA}] \end{array}$$

Here the asterisk (^∗^) indicates the activated or phosphorylated form of the enzyme. Here and below, PKA refers the catalytic subunits (Tpk1, Tpk2, or Tpk3). Equations (1)–(3), below, model the velocities of these reactions using an extension of Michaelis-Menten kinetics [51] that allows for competition between Pde1, Pde2 and Ira for PKA.

(1) $$\frac{d}{\text{dt}}[\text{Pde}1^{*}]:\frac{R_{p_{1}}[\text{Pde}1][\text{PKA}]}{\Gamma_{p1}+[\text{Pde}1]+\frac{\Gamma_{p1}}{\Gamma_{p2}}[\text{Pde}2]+\frac{\Gamma_{p1}}{\Gamma_{z}}[\text{Ira}]}$$

(2) $$\frac{d}{\text{dt}}[\text{Pde}2^{*}]:\frac{R_{p_{2}}[\text{Pde}2][\text{PKA}]}{\Gamma_{p2}+[\text{Pde}2]+\frac{\Gamma_{p2}}{\Gamma_{p1}}[\text{Pde}1]+\frac{\Gamma_{p2}}{\Gamma_{z}}[\text{Ira}]}$$

(3) $$\frac{d}{\text{dt}}[{\text{Ira}}^{*}]:\frac{R_{z}[\text{Ira}][\text{PKA}]}{\Gamma_{z}+[\text{Ira}]+\frac{\Gamma_{z}}{\Gamma_{p1}}[\text{Pde}1]+\frac{\Gamma_{z}}{\Gamma_{p2}}[\text{Pde}2]}$$

In order for the model to replicate the dynamics observed by Ma et al. [35] (Figure 2) we impose the following conditions: 

Condition (a) The following inequalities must hold:

$$\frac{\Gamma_{p1}[\text{Pde}2]}{\Gamma_{p2}[\text{Pde}1]}\ll 1$$

$$\frac{\Gamma_{p2}[\text{Ira}]}{\Gamma_{z}[\text{Pde}2]}\ll 1$$

Condition (b) In comparing analogous reactions of Pde1 and Pde2, the reactions of Pde2 are uniformly slower.

Condition (c) PKA rapidly phosphorylates Ira.

By invoking Condition (a), the effect of Pde2 is negligible when Pde1 is active. This assumption is justified by the observation that the dynamics of cAMP signaling following glucose stimulus are essentially unchanged in the *pde2Δ* mutant. By invoking Condition (b) we can model both Pde1 and Pde2 with a single equation (for details and derivation see the Additional file 1: Supplementary Model). Condition (b) is justified by the large transient cAMP signal observed in the *pde1Δ* mutant. Neither Condition (a) nor (b) are required to reproduce the behavior of the wild-type or mutant strains, but are mathematically convenient for exploring the model analytically as described below.

Condition (c) reflects negative feedback on the Ras branch of the pathway. We impose Condition (c) so that even when PKA’s phosphorylation of Ira is slowed by the presence of either Pde1 or Pde2, by Condition (a), the effect of PKA on Ira can not be ignored. In the double mutant case (*pde1Δpde2Δ*) the loss of competitive inhibition of PKA greatly enhances PKA’s effect on Ira. Thus in the double mutant case PKA has a strong negative effect on Ras ·GTP. Several studies have provided experimental support for a role of the Ira proteins in negative feedback on cAMP signaling [21,22] as well as evidence that this feedback is regulated by PKA [52].

### Steady state assumptions

The model may be further simplified by assuming that the following four reactions are fast and hence proceed to steady state. 

1. Gpa2 ·GDP ⇌ Gpa2 ·GTP (reaction 1 in Figure 1).

2. Activation/inactivation of Cdc25 (reaction 2 in Figure 1).

3. Activation/inactivation of adenylate cyclase (reaction 5 in Figure 1).

4. cAMP+[Bcy1::PKA] ⇌ [cAMP::Bcy1] +PKA (reaction 9 in Figure 1).

Again, these steady state assumptions are made for mathematical simplicity, and are not a requirement to mathematically reproduce wild-type or mutant signaling dynamics. We will only focus on the last of these steady state assumptions, for the rest we refer the reader to the Additional file 1: Supplementary Model. We assume that PKA activates rapidly, that is we assume that the concentration of active PKA can be treated as being at steady state. This assumption was taken for mathematical simplicity of the model, it is not a necessary condition to numerically replicate the dynamics observed by either Ma et al. or Garmendia-Torres et al. In our model PKA feedback plays a central role, thus we will examine this steady state condition in detail. In the cAMP-PKA pathway PKA is activated by cAMP in the following manner: four cAMP molecules bind to two regulatory subunits (Bcy1) and release two catalytic subunits, creating the active form of PKA.

$$\;4[\text{cAMP}]\;+[2\text{Bcy1}::\;\text{PKA}]\overset{k_{b}}{\underset{k_{f}}{⇌}}[4\text{cAMP}\;::2\text{Bcy1}]\;+[2{\text{PKA}}^{*}]$$

When modeling this reaction we can simplify this in two ways: 1) by assuming that the concentration of active PKA does not approach its maximum; and 2) by approximating the 4 to 2 ratio by a 2 to 1 ratio. Applying this simplification we can model the change in PKA activity as proportional to cAMP squared (*x*^2^); thus in Equations (4a)–(4d) (below) *x*^2^ represents PKA feedback (for full details see the Additional file 1: Supplementary Model).

### Simplified model

Combining feedback Conditions (a), (b), and (c) with the four steady state assumptions, and by modeling both forms of PDE with a single case dependent variable, *p*, we reduce the nine-dimensional model shown in Figure 1 to the simpler four-dimensional model depicted in Figure 3. The model in Figure 3 is described by the following system of equations:

(4a) $$\begin{array}{l} \text{Ras}\;:\;\frac{\text{dr}}{\text{dt}}=\frac{A(1-r)}{\Gamma_{1}+1-r}-\frac{\text{Bzr}}{\Gamma_{1}+r}\; \end{array}$$

(4b) $$\begin{array}{l} \text{Ira}\;:\;\frac{\text{dz}}{\text{dt}}\;=\;N(x^{2}-z)\; \end{array}$$

(4c) $$\begin{array}{l} \text{Pde}\;:\;\frac{\text{dp}}{\text{dt}}=M(x^{2}-p)\; \end{array}$$

(4d) $$\begin{array}{l} \text{cAMP}\;:\;\frac{\text{dx}}{\text{dt}}=C+\text{Gr}-D_{0}x-\frac{\text{Dpx}}{\Gamma +x}\; \end{array}$$

In Equations (4a)–(4d) and in Figure 3 the concentration of Ras ·GTP is represented by variable *r*; the concentration of Ras ·GDP is represented by (1−*r*); the concentrations of Ira1 and Ira2 are represented by *z*; the variable *p* represents the concentration of Pde1 in Cases 1, 3, and 4 (*wt*, *pde2Δ*, and *pde1*^*ala*152^) and the concentration of Pde2 in Case 2 (*pde1Δ*); the concentration of cAMP is represented by *x*; as stated above *x*^2^ represents the concentration of active PKA; and time is given by variable *t*. All variables have been nondimensionalized. The constants in Equations (4a)–(4d) are nondimensional composites of dimensional parameters of the full system, derived in the Additional file 1: Supplementary Model. Intuitively, *A* is the activation rate of Ras ·GTP catalyzed by Cdc25; *B* is the inactivation rate of Ras ·GTP catalyzed by Ira1 and Ira2; *Γ*_1_ is the affinity of both Cdc25 and Ira1/2 for Ras ·GTP; *N* is the reaction rate of Ira1 and Ira2; *M* is the quantitative expression of Condition (b); *C* is the production rate of cAMP due to the basal activity of adenylate cyclase; *G* accounts for the glucose, normalized so that *G*=1 after a glucose stimulus is applied; *D*_0_ represents a “basal” decay rate of cAMP in the absence of activated Pde; *D* represents enhanced decay due to PKA feedback on Pde modified by *Γ*, the affinity of activated Pde for cAMP.

## Results

In this section we examine analytical, numerical, and experimental results motivated by our model of the cAMP-PKA pathway. We show that the simplified version of the model, given by Equations (4a)–(4d), can adequately replicate the short-term dynamics of cAMP, reported by Ma et al. [35] for wild-type cells and all three phosphodiesterase mutants (*pde1Δ*, *pde2Δ*, *pde1Δpde2Δ*). For the wild-type case we analyze the behavior of the system as it approaches steady state and find that for a wide range of parameters the model predicts that cAMP levels should exhibit decaying oscillations. Since oscillatory behavior was not an explict input into our modeling effort, we considered this a novel prediction, and undertook an experimental validation of the model using time series measurements of cAMP signaling for two diploid yeast strains. As predicted by the mathematical model we observed decaying cAMP oscillations following glucose stimulation. We then extended the model to consider longer-time scale, sustained cAMP oscillations (5-10 minute periods) in response to nutrient stress, as predicted by Garmendia-Torres et al. [42]. We show that the core model can also reproduce sustained oscillations on this longer time scale, and we discuss the model parameters and the corresponding biochemical interactions, that are required to generate such sustained oscillations.

### Short-term dynamics

**Figure 4:**
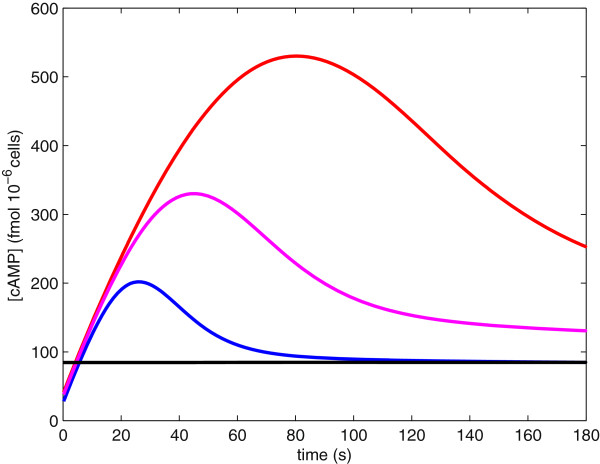
**Numerical simulations under the simplified model Equations (4a)–(4d), fit to the data of Ma et al(Parameters as given in Tables**1** and **2**).** The corresponding genotypes are as in Figure 2. The *wt* and *pde2Δ* cases overlap each other in this figure.

Our first task is to demonstrate that Equations (4a)–(4d) can adequately replicate the dynamics of cAMP signaling immediately following glucose stimulation, for both wild-type cells and mutants lacking either one or both phosphodiesterases. Figure 4 shows the modeled concentration of cAMP as a function of time, following glucose stimulation of wild-type and PDE mutants. This outcome was generated using the parameter choices given in Tables 1 and 2. The dynamical patterns generated by the model are a good match to those illustrated in Figure 2. The dimensional concentrations in the figure were obtained by multiplying *x* in Equations (4a)–(4d), the dimensionless variable representing cAMP concentrations, by 24.95 fmol·10^−6^ cells; and multiplying time, given by dimensionless variable *t*, by.038 minutes. These scale factors emerge from the discussion in the Additional file 1: Supplementary Model. Initial conditions were the steady-state concentrations that occur under glucose starved conditions.

**Table 1:** Parameter values that vary when fitting Equations (4a)–(4d) to the Ma et al. data

**Parameter**	***wt***	***pde1Δ***	***pde2Δ***	***pde1Δ***^***ala152***^	***pde1Δ*** ***pde2Δ***
*M*	0.01	0.0005	0.01	0.0025	–
*D*	1	0.26	1	0.54	–
*Γ*	33.6	16.8	33.6	33.6	–
*B*	.0051	.0051	.0051	.0051	.51

Here *M* is the quantitative expression of Condition (b) [see text]; *D* represents enhanced decay due to PKA feedback on Pde modified by *Γ*, the affinity of activated Pde for cAMP; *B* is the inactivation rate of Ras ·GTP due to Ira1/2. Also see the text for a discussion of the great overlap between columns 2 and 4.

**Table 2:** Parameter values that are consistent when fitting Equations (4a)–(4d) to the Ma et al. data

**Parameter**	**Value**
*A*	1.45
*Γ*_1_	0.0004
*N*	0.032
*C*	0.044
*D*_0_	0.013

Here *A* is the activation rate of Ras ·GTP catalyzed by Cdc25; *Γ*_1_ is the affinity of both Cdc25 and Ira1/2 for Ras ·GTP; *N* is the reaction rate of Ira1/2; *C* is the production rate of cAMP due to the basal activity of adenylate cyclase; *D*_0_ represents the decay rate of cAMP in the absence of activated PDEs.

Note that the numbers in columns of Table 1 that represent the wild-type and *pde2Δ* backgrounds are identical. Because of Condition (a) above, when Pde1 is present, the effect of Pde2 is negligible. Thus, the values in these columns of the table characterize properties of Pde1. Similarly, the values in the *pde1Δ* column characterize properties of Pde2. In the case of *pde1*^*ala*152^ both the activation and inactivation rate of Pde1 decrease, but the affinity of Pde1 for cAMP (*Γ*) does not change. The value of *B* is increased in the double PDE knockout because, by Condition (a), when both forms of PDE are eliminated the effect of PKA on Ira1/2 is greatly enhanced. As mentioned above, the dimensionless parameters in Equations (4a)–(4d) are composites of dimensional parameters. In choosing dimensional parameters we used values given by Garmendia-Torres et al. [42] when available. Moreover, the parameters determined by fitting the model also lie in biologically appropriate ranges. We refer to the Additional file 1: Supplementary Model for the details of the fitting process.

An intuitive explanation for the behavior of wild-type cells is that the transient cAMP peak is due to the delay between the increase of adenylate cyclase activity and PKA mediated enhancement of phosphodiesterase activity. Activation of adenylate cyclase activity increases cAMP concentrations, leading to the release of PKA catalytic subunits. PKA in turn activates the phosphodiesterases, which enhances the decay of cAMP and brings the concentration of cAMP down to its steady state level. In wild type cells, we propose that Pde1 is the primary effector of PKA feedback. In the *pde1Δ* and *pde1*^*ala*152^ cases the slower activation of Pde2 accounts for the greater transient observed in these cases.

### Oscillatory approaches to steady state

For a wide range of parameters the model described by Equations (4a)–(4d) suggests that cAMP concentration should exhibit decaying oscillations as it approaches its steady state value following glucose stimulus. Here we describe the conditions required to generate this behavior.

#### Decaying cAMP oscillations via feedback on PDEs

PKA feedback through the PDEs alone is sufficient to generate decaying cAMP oscillations following glucose stimulus. To see this we examine the case when Ras is either completely in the Ras ·GTP or Ras ·GDP state. When *A* is sufficiently large, or sufficiently small, we can reduce the model by making the approximation that the concentration of Ras stays completely either in the GTP-bound (*r*_*ss*_≈1), or GDP-bound form (*r*_*ss*_≈0). This reduces our model, Equations (4a)–(4d), to a two-by-two system, representing change in concentration of Pde (*p*) and cAMP (*x*):

(5a) $$\frac{\text{dp}}{\text{dt}}=M(x^{2}-p)$$

(5b) $$\frac{\text{dx}}{\text{dt}}=C_{0}-D_{0}x-\frac{\text{Dpx}}{\Gamma +x},$$

where *C*_0_=1+*C* when Ras ·GTP is the dominant Ras form (*r*_*ss*_≈1) or *C*_0_=*C* when Ras ·GDP dominates (*r*_*ss*_≈0). In these cases we can find an explicit expression for when the concentration of cAMP exhibits decaying oscillation as it approaches steady state.

##### Claim 1

Solutions of (5a) and (5b) exhibit an oscillatory approach to steady-state if and only if

(6) $${\left(M-D_{0}-\frac{\mathrm{\Gamma D}x_{\text{ss}}^{2}}{{\left(\Gamma +x_{\text{ss}}\right)}^{2}}\right)}^{2}<8M\frac{Dx_{\text{ss}}^{2}}{\Gamma +x_{\text{ss}}}.$$

where *x*_*ss*_ is the equilibrium solution of (5a, 5b); i.e.,

(7) $$C_{0}-D_{0}x_{\text{ss}}-\frac{Dx_{\text{ss}}^{3}}{\Gamma +x_{\text{ss}}}=0.$$

**Figure 5:**
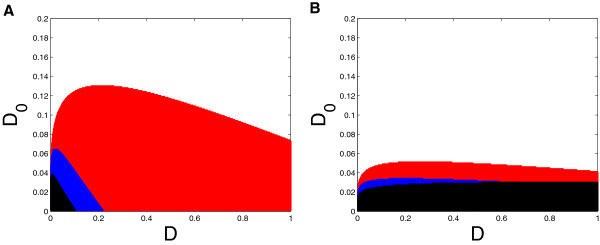
**Solutions to the inequality given in Equation (6) for two different values of the model parameter *C*. A**) *C*_0_ = 1.044; **B**) *C*_0_ = .044. The overlapping shaded regions of these figures illustrate the parameter ranges for *D* and *D*_0_ where the model predicts the possibility of cAMP oscillation. The black region is *Γ* = 33.6, the *Γ* value for Ma et al. *wt*; the blue region is *Γ* = 3.36; and the red region is *Γ* = .336. In both cases, *M* = .01.

Condition (6) is derived by computing the Jacobian matrix of Equations (5a) and (5b) at the equilibrium point and determining when the eigenvalues have a nonzero imaginary part. Because of the implicit definition of *x*_*ss*_, it is rather difficult to apply directly. Therefore we refer to Figures 5a and 5b, in which the overlapping shaded regions show the ranges of *D*_0_ and *D* for which the inequality (6) is satisfied, for several values of *Γ* and for *M* assumed equal to.01. Figure 5a shows the parameter range for *C*_0_=1+*C*=1.044, the case when *r*_*ss*_≈1, and Figure 5b shows the parameter range for *C*_0_=*C*=.044, the case when *r*_*ss*_≈0. Observe that the value of *Γ* has a large effect on when oscillation occurs: when *Γ* is small (i.e. the PDEs have high affinity for cAMP), oscillations occur over a large parameter range, but as *Γ* increases, the parameter range decreases significantly. Finally, because of the factor of *M* on the right-hand side of (6), it is difficult to satisfy the inequality if *M* is very small; thus decaying oscillations are less likely for the *pde1Δ* and *pde1*^*ala*152^ mutants.

#### General conditions for decaying oscillations

We now seek to understand the parameters that cause decaying cAMP oscillations when Ras activation is free to vary. To do so we numerically examine when the Jacobian matrix determined by the four–by–four system has a complex conjugate pair of eigenvalues with negative real parts. We summarize the results here. We find that as parameters *Γ*_1_, *N* and the ratio $\frac{A}{B}$ increase we are more likely to observe decaying oscillations. We also find that as the parameters that enhance the decay of cAMP are increased, that is as parameters *D*_0_ and *D* are increased or as *M* decreases decaying oscillations are less likely. For a more detailed analysis we refer the reader to the Additional file 1: Supplementary Model.

### Experimental validation of predicted cAMP oscillations

Our model makes the novel prediction that for many choices of parameter values, cAMP levels should exhibit decaying oscillations towards a steady-state following glucose stimulus. In order to test this hypothesis we measured the dynamics of the cAMP response in diploid cells of two strains of *S. cerevisiae* – S288c and *Σ*1278b. S288c is considered the standard “reference” genome for yeast studies while *Σ*1278b is commonly used for studies of developmental pathways in yeast [53,54].

**Figure 6:**
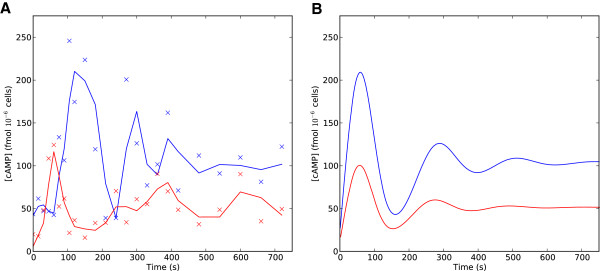
**Variation in cAMP signaling between strain backgrounds. A**) Experimental time series of cAMP concentration following glucose stimulus for S288c (red) and *Σ*1278b (blue). x’s indicate measured cAMP values, the solid lines indicate smoothed values fit by convolving the data with a Blackman window kernel filter. **B**) Numerical simulations under the non-dimensional model to fit *Σ*1278b (blue) and S288c (red).

For each strain we monitored cAMP levels for eight to twelve minutes following a glucose stimulus. Two typical experimental time series are illustrated in Figure 6. There are cAMP oscillations in both genetic backgrounds but the quantitative features of the oscillations appear to be strain dependent. *Σ*1278b exhibits a classical form of decaying oscillations characterized by a large cAMP peak immediately after the stimulus, and dampening oscillations towards a new steady state. In the S288c background, by contrast, the oscillations appear to be delayed, occur with a lower amplitude and do not decay as rapidly. The *Σ*1278b strain has a cAMP peak approximately twice as large as that of S288c.

There are various ways in which we could fit the dynamics reported here. We could assume that Ras ·GTP is saturated, as in Ma et al. Cases 1–4 and seek to fit only the two–by–two system, given by Equations (5a) and (5b), to this data. Alternatively we could fit all four equations, (4a)–(4d), to this data. Since this experiment was done in a similar way to the Ma et al. [35] experiment, we seek to fit the data to parameters relating to only Pde feedback, that is parameters *D*, *Γ*, and *M*. The data is taken in increments of fifteen seconds for the first two minutes, thirty seconds for the next five minutes and every minute for the last five minutes. We used a least squares approach to fit the parameters of the mathematical model to the observed data. Because the density of samples is higher in the initial two minutes, the fitting more closely resembles the data over this interval.

We can approximate the dynamics observed for both *Σ*1278b and S288c using the model described by Equations (4a)–(4d) (Figure 6A). Table 3 shows the parameter values used to fit the oscillations in *Σ*1278b and S288c compared to the parameter values used to fit Ma et al. wild type. For both cases the model requires Pde’s affinity for cAMP, $\frac{1}{\Gamma }$, to be much greater than Pde’s affinity for cAMP in the Ma et al. case. In our fitting we note that the most significant difference between the two strains is in the decay of cAMP with respect to Pde (parameter D).

**Table 3:** **Parameter values used to replicate cAMP dynamics for strains *Σ *1278b and S288c (both wild types) compared to parameter values used to fit the wild type data reported in Ma et al. [**[35]**]**

**Parameter**	**Interpretation**	**Ma**	***Σ*1278b**	**S288c**
*M*	Rate at which Pde activity goes to steady state	.01	.085	.19
*D*	Decay rate of cAMP due to active Pde1	1	0.247	1.07
*Γ*	Pde1 affinity for cAMP	33.6	0.32	0.15
*T*	Time scale	.0377	.17	.35

#### PDE mutants in the *Σ*1278b background

**Figure 7:**
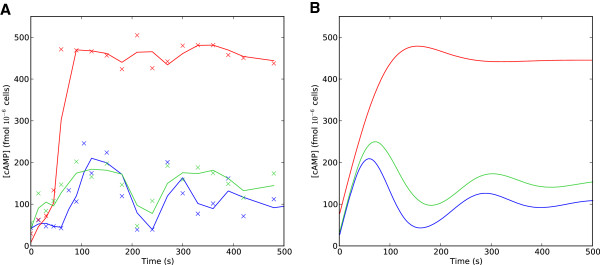
**The signaling behavior of PDE mutants in the *Σ *1278b strain background. A**) Experimental time series of cAMP concentration following glucose stimulus for *Σ*1278b wild-type (blue), *pde1Δ* (red) and *pde2Δ* (green) strains. x’s indicate measured cAMP values, the solid lines indicate smoothed values, as in Figure 7. **B**) Numerical simulations under the non-dimensional model to fit to the observed data.

The *Σ*1278b wild-type strain exhibited a considerably larger cAMP peak and more pronounced oscillations than S288c (Figure 6), or the the W303 backround used by Ma et al. We therefore undertook additional experiments to examine the behavior of the *pde1Δ* and *pde2Δ* knockout mutants in *Σ*1278b. Typical experimental time series for the PDE mutants in the *Σ*1278b background are shown in Figure 7. These experiments qualitatively agree with the dynamics reported by Ma et al. for the W303 background: 1) the *pde2Δ* mutant has very similar cAMP dynamics to the wild-type background; and 2) the *pde1Δ* mutant reaches a much higher cAMP maximum. One notable difference between the W303 and *Σ*1278b *pde1Δ* mutants is that cAMP levels in the *Σ*1278b background reach a steady-state at or near their maximum, while the W303 *pde1Δ* mutant shows a more transient response with cAMP levels decaying towards a steady state significantly lower than the maximum. Because of this observed difference in the cAMP signaling behavior, when fitting model parameters for the *pde1Δ* and *pde2Δ* mutants we relaxed Condition (b), meaning that we no longer assumed a uniformly slower rate of activation/inactivation for Pde2. Model parameter estimates for the *Σ*1278b PDE mutants are given in Table 4. Comparing the parameters of the *pde1Δ* mutants in W303 (Table 1) and *Σ*1278b (Table 4), we see that the non-dimensional parameter *M*, representing the rate at which phosphodiesterase activity reaches its steady state, increases relative to the wild-type in the *Σ*1278b background, reflecting the lack of a transient cAMP response in this strain. The difference between the backgrounds in terms of steady state cAMP levels following stimulus is primarily reflected in the relative change in the parameter *D*.

**Table 4:** **Parameter values used to replicate cAMP dynamics for the phosphodiesterase mutants, *pde1 Δ * and *pde2 Δ * in the *Σ *1278b background**

**Parameter**	***wt***	***p d e *1*Δ***	***p d e *2*Δ***
*M*	0.085	0.262	0.121
*D*	0.247	0.0112	0.107
*Γ*	0.32	0.04	0.061

For all three cases, the time scale parameter *T* =0.17.

### Long-term dynamics

**Figure 8:**
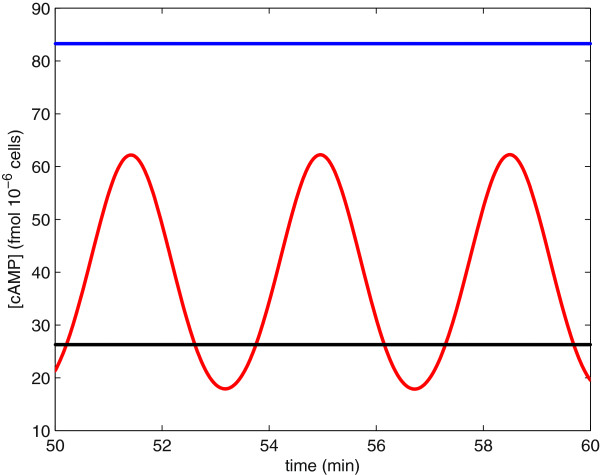
**Long-term dynamics for the concentration of cAMP.** The blue line shows the cAMP concentration at high nutrient levels and/or low stress levels (parameter *A* = 1.4). The black line shows the concentration of cAMP at low nutrient levels and/or high stress levels (parameter *A* = .005). The red curve shows the concentration of cAMP at intermediate nutrient levels and/or intermediate stress levels (parameter *A* = .014).

If we consider our model over time scales longer than five minutes, our model the cAMP-PKA pathway predicts that cAMP levels may either go to a unique steady state or experience sustained oscillations. Garmendia-Torres et al. [42] predicted sustained oscillations in the concentration of cAMP at intermediate stress levels. Since stress affects our model through parameter *A* (the activation rate of Ras ·GTP), we choose *A* as our bifurcation parameter. At intermediate stress levels, for example *A*=.014, the long-term concentration of cAMP experiences sustained oscillations, as seen by the red curve in Figure 8. When stress levels are low, for example *A*=1.4, the long-term concentration of cAMP remains at a high steady-state value, as seen by the blue line in Figure 8. When stress levels are high, for example *A*=.005, the long-term concentration of cAMP remains at a low steady-state value, as seen by the black line in Figure 8.

An intuitive biological explanation of this behavior is that at intermediate stress levels Ras (*r*) alternates between active (GTP-bound, *r*_*ss*_≈1) and inactive (GDP-bound, *r*_*ss*_≈0) states. This oscillation in Ras states causes oscillations in the concentration of cAMP. When stress levels are low, for example in the glucose fed state, Ras is almost completely in the Ras ·GTP state, as in Ma et al. [35] Cases 1–4, forcing the long-term concentration of cAMP to steady state. Similarly, when stress levels are high, Ras is primarily in the Ras ·GDP state, as in Ma et al. [35] Case 5, forcing the long-term concentration of cAMP to a low steady state.

#### Analysis of sustained oscillations

Analysis of our model suggests that sustained oscillations are the result of PKA feedback through the Ras pathway (variables *r* and *z*). In contrast, negative feedback through the PDEs (variable *p*) stabilizes the system. This analysis was done by examining the stability of the system by evaluating the Jacobian matrix at the equilibrium. We find that our system loses stability through a Hopf bifurcation; thus, indicating that the system experiences sustained oscillations (for details see Additional file 1: Supplementary Model). This instability, leading to sustained oscillations, is a result of Ras varying between its active (GTP) and inactive (GDP) states. Activation/inactivation of Ras is controlled by parameters *A*, *B*, and *Γ*_**1**_ in our model. We find that as the value of *Γ*_**1**_ increases our system is less likely to experience sustained oscillations. Oscillations are also more likely as the ratio $\frac{A}{B}$ increases. In regards to the period and amplitude of oscillations, we observe that as both forms of PKA feedback (through Ras and PDE) slow down, that is as the values of parameters *N* and *M* decrease, the length of the period of oscillation increases. Finally, we conclude that as the parameters that enhance the decay of cAMP are decreased, that is as parameters *D*_**0**_ and *D* are decreased and parameter *Γ* is increased, oscillations are more likely to have longer periods and larger amplitudes.

## Discussion

The model presented here is, to the best of our knowledge, the first analytical model of the yeast cAMP-PKA pathway that is capable of recapitulating both the short-term and long-term dynamics of cAMP signaling in wilde-type cells. The model we propose is also capable of replicating cAMP dynamics that have been observed single and double phosphodiesterase mutants. In addition to capturing behaviors previously described our model makes new predictions about cAMP oscillations. Our work also highlights variation in cAMP signaling between genetic backgrounds.

### cAMP oscillations

Analysis of a non-dimensional system derived from our model suggests that a substantial fraction of the model parameter space for the wild-type signaling network should result in decaying cAMP oscillations following glucose stimulus. While long time-scale oscillations have previously been predicted [39,42], short-term oscillations, on the scale of minutes, have not been noted in the yeast literature. To test our predictions we experimentally measured the cAMP response in two different diploid strains of *S. cerevisiae* and observed oscillations as predicted by the model. While ours is the first study to document short time-scale cAMP oscillations in yeast, such oscillations have been noted in other systems including mammals, amphibians, and slime molds [55-57]. For example, Dyachock et al. [55] reported short time-scale glucose induced oscillations in mouse pancreatic *β*-cells. They showed that these oscillations correlated with pulsatile insulin release. Similar oscillations are observed in response to stimulation of such cells by the peptide hormone GLP-1 [58]. The slime mold *Dictyostelium* also exhibits short time-scale cAMP oscillations [57]. Similar to the model we present here, Maeda et al. [57] suggested that positive feedback by PKA on phosphodiesterase activity and PKA negative feedback on adenylate cyclase activity are critical features of the molecular circuit that generates these oscillations in *Dictyostelium*. Whether cAMP oscillations in these diverse eukaryotic groups are due to conserved interactions in the cAMP pathway or whether such dynamics represent a convergent feature of cAMP signaling is a key question for future comparative studies.

Our model is also able to generate longer-time scale cAMP oscillations. Such oscillatory dynamics should be reflected in PKA activity. Recent studies [59,60] have demonstrated that PKA activation plays an important role in regulating oscillatory patterns of nuclear localization of stress-responsive transcription factors such as Msn2. Different dynamical patterns of Msn2 regulation in turn can lead to qualitatively different expression outputs of downstream targets [59].

### Comparison to other models

There have been a number of recent studies that have proposed mathematical models for the cAMP-PKA network in yeast, including Gonze et al. [39], Cazzaniga et al. [40], and Williamson et al. [41]. A limitation shared by all three of these models is that they can not recapitulate the dynamical behaviors of the double phosphodiesterase mutant (*pde1Δpde2Δ*). This can be seen by setting the variables representing the phosphodiesterases to zero in any of these models (i.e. representing the double knockout); the result is a rapid accumulation of cAMP. This contrary to the observed data reported by Ma et al. [35]. Our model uses competitive inhibition between Pde1, Pde2 and the GTPase activating proteins Ira1 and/or Ira2 to explain the double PDE knockout case; thus, competitive inhibition between Pde1, Pde2, and Ira1/2 is what sets our model apart from these previous models.

Gonze et al. [39] propose a stochastic model, that is a direct extension of the Garmendia-Torres et al. model [42], to explore the effects of the cAMP-PKA pathway on the transcription factor Msn2. This model only focuses on the long term dynamics and can not be used to understand any of the PDE mutant cases proposed by Ma et al. Cazzaniga and colleagues [40] employed a stochastic modeling approach to study the yeast cAMP-PKA network. Though they do explore the effects of different PDE activities, their model does not explicitly account for the mutant cases described by Ma et al. and again they can not replicate the double phosphodiesterase knockout. Also they do not consider the long term dynamics proposed by Garmendia-Torres et al. [42]. The deterministic model presented by Williamson et al. [41] is the closest in approach to ours. Their model is able to recapitulate the relevant dynamics of the Pde1 and Pde2 single mutants but does not account for the behavior of the PDE double mutant. As we mentioned above this is significant since the double knockout case is the most surprising case and the hardest case to explain. They also only examined the short term dynamics of cAMP, where as our model accounts for both the long and short term dynamics of cAMP.

### Conclusions

The mathematical model we present here replicates the dynamical behavior of wild-type and mutant cells and leads us to novel predictions of oscillatory cAMP dynamics in *S. cerevisiae*. Experiments motivated by this modeling effort confirmed these predictions and also highlighted variation in oscillatory phenomena among yeast isolates. The mathematical model, in turn, is capable of reproducing the different signaling phenotypes we observed and allows us to predict which parts of the network are most likely to contribute to signaling variation. Because it has both predictive and explanatory power this model can serve as a foundation for future mathematical and experimental explorations of cAMP signaling. A future challenge for both experimental and modeling studies of cAMP signaling will be to determine whether the grossly similar oscillatory dynamics observed across eukaryotes is due to a common mechanistic basis or whether this represents an example of convergent evolution.

## Methods

### Strains and cAMP assay

Diploid strains of the laboratory backgrounds *Σ*1278b (G85 [‘wild-type’, ura3 *Δ*0, his3 *Δ*0::hisG], G30025 [‘*pde1Δ*’, pde1::bcKanMX4, ura3 *Δ*0, his3 *Δ*0::hisG], G30025 [‘*pde2Δ*’, pde2::bcKanMX4, ura3 *Δ*0, his3 *Δ*0::hisG], gifts of T. Galitski [61]) and S288c (BY4743, gift of D. Lew) were used to study cAMP dynamics. Cells were prepared following a protocol adapted from Paiardi et al. [17]. Approximately 2×10^9^ cells/ml were grown in rich media (1% yeast extract, 2% peptone and 2% glucose) at 30°C and transferred to SC media with 0.1% glucose and 3% gycerol for overnight incubation at 30°C. Cells were collected, washed and incubated in 25 mM MES buffer, pH 6 (Boston Bio Products, Worcester, MA) for 30 minutes at 30°C before glucose induction. Glucose was added to 2 ml of cell culture(approx. 2 ×10 ^8^ cells/ml) to a final concentration of 100 mM and 25 mM. After glucose induction cells were collected over a 12-minute time course in 15 second increments for the first minute and at 30 second increments for the rest of the time course. 3 ×10 ^6^ cells were collected at each time point, fixed in 300 *μ*l n-butanol-saturated 1M formic acid (Jiang et.al., 1998) and frozen at -80°C. Cells were lysed using four freeze-thaw cycles and freeze-dried in a Speed Vac concentrator (Savant Instruments, Farmingdale, NY). Cell extracts were used to determine cAMP concentration using a cAMP Biotrak Enzyme immunoassay kit (Amersham, GE Healthcare) and a SpectraMax spectrophotometer (Molecular Devices, Sunnyvale, CA).

### Model fitting

For the purposes of model fitting, where available we used estimates of key biochemical parameters as reported in [42]. In cases where such parameters were not avaiable, we used a method of least squares, implemented in the subroutine lmdif.f in the Fortran MINPACK library [62]. This subroutine minimizes the sum of the squares of *m* nonlinear functions in *n* variables by a modification of the Levenberg-Marquardt algorithm. We wrote a C computer program that calculates the appropriate functions that are used by this algorithm for the model described above. Additional details of the model fitting procedure are provided in the Additional file 1: Supplemental materials.

## Competing interests

The authors declare that they have no competing interests.

## Authors’ contributions

KG carried out the mathematical modeling. OK conducted biochemical measurements of cAMP signaling. DGS contributed to the mathematical modeling. PMM contributed to design of the study and carried out analysis of the experimental data. All authors participated in the drafting of the manuscript, and have read and approved the final manuscript.

## Supplementary Material

Additional file 1 **Supplementary model **[35,42,62,63]**.** Click here for file
