# Supplementary material for: Modeling mutant phenotypes and oscillatory dynamics in the Saccharomyces cerevisiae cAMP-PKA pathway
Source: BMC Syst Biol. 2013 May 17;7:40. doi: 10.1186/1752-0509-7-40 (PMC3679983; doi:10.1186/1752-0509-7-40)
Supplement: Additional file 1 — Supplementary model [35,42,62,63]. [file 1752-0509-7-40-S1.pdf]

Additional Files 1: Supplementary Model

S1 Full Model

To fully describe the reactions in Figure 1 a system of nine time-dependent differential equations is needed. This system models the change in the concentrations of: 1) Gpa2·GTP (active Gpa2); 2) Cdc25; 3) Ras·GTPase Ira1 and Ira2; 4) Ras·GTP (active Ras2); 5) Adenylate cyclase activity; 6) activated Pde1; 7) activated Pde2; 8) cAMP; and 9) active PKA (free catalytic subunits). The concentrations have units of fmol/(10^6 cells) and time has scale of minutes.

[Gpa2·GTP] $$\frac{dg}{dt} = P_g[\text{Glu}] - D_g g \tag{S1}$$

[Cdc25*] $$\frac{dc}{dt} = P_c - D_c[\text{Stress}]c \tag{S2}$$

[Ira*] $$\frac{dz}{dt} = \frac{R_z(C_z - z)y}{\Gamma_z \sum(z, p_1, p_2)} - D_z z \tag{S3}$$

[Ras·GTP] $$\frac{dr}{dt} = \frac{R_r c(C_r - r)}{\Gamma_r + C_r - r} - \frac{\bar{R}_r zr}{\Gamma_r + r} \tag{S4}$$

[Adenylate cyclase] $$\frac{da}{dt} = (P_a + \bar{P}_a gr) - D_a a \tag{S5}$$

[cAMP] $$\frac{dx}{dt} = P_x a - \left(D_x x + \frac{R_{x1} p_1 x}{\Gamma_{x1} + x} + \frac{R_{x2} p_2 x}{\Gamma_{x2} + x}\right) - 4\left(K_b x^4 (C_y - \frac{y}{2}) - K_f y^3\right) \tag{S6}$$

[Pde1*] $$\frac{dp_1}{dt} = \frac{R_{p_1}(C_{p_1} - p_1)y}{\Gamma_{p_1} \sum(z, p_1, p_2)} - D_{p_1} p_1 \tag{S7}$$

[Pde2*] $$\frac{dp_2}{dt} = \frac{R_{p_2}(C_{p_2} - p_2)y}{\Gamma_{p_2} \sum(z, p_1, p_2)} - D_{p_2} p_2 \tag{S8}$$

[PKA] $$\frac{dy}{dt} = 2\left(K_b x^4 (C_y - \frac{y}{2}) - K_f y^3\right) \tag{S9}$$

Here the asterisk (*) indicates the activated form of the enzyme. If an equation has two similar reactions, we use a bar over the second parameter to indicate that this is a different parameter but of a similar type to the first parameter.

The notation for the parameters is as follows: P is a production term at a rate independent of the concentration of the enzyme being produced; D is a linear decay rate; K is a reaction coefficient in a binary (or higher order) reaction; R is a reaction coefficient in a catalyzed reaction (that is the Michaelis-Menten rate); C is a total concentration of an enzyme; and Γ is a Michaelis-

Menten affinity. In Equations (S3), (S7) and (S8),

$$\sum(c, p_1, p_2) = 1 + \frac{C_z - z}{\Gamma_z} + \frac{C_{p_1} - p_1}{\Gamma_{p_1}} + \frac{C_{p_2} - p_2}{\Gamma_{p_2}}$$

To simplify (S3), (S7), and (S8), assume that the total concentrations of Ira, Pde1, and Pde2 (C_z, C_{p_1}, and C_{p_2}) are always much greater than the active concentrations of Ira, Pde1, and Pde2. Thus, we may approximate inactive Pde1 ([PDE1] in (1)) as C_{p_1}. We may also approximate inactive Pde2 ([PDE2] in (2)) as C_{p_2}, and approximate inactive Ira ([Ira] in (3)) as C_z. That is $C_z - z \approx C_z$, $C_{p_1} - p_1 \approx C_{p_1}$, and $C_{p_2} - p_2 \approx C_{p_2}$ in Equations (S3), (S7) and (S8).

S2 Simplification of the Full Model

As noted in Section 3.1, in order for the model to replicate the dynamics observed by Ma et al. [35] (Fig. 2), the following three conditions are essential.

Condition (a) The following inequalities must hold:

$$\frac{\Gamma_{p_1} C_{p_2}}{\Gamma_{p_2} C_{p_1}} << 1 \tag{S10}$$

$$\frac{\Gamma_{p_2} C_z}{\Gamma_z C_{p_2}} << 1 \tag{S11}$$

Condition (b) In comparing analogous reactions of Pde1 and Pde2, the reactions of Pde2 are uniformly slower.

Condition (c) PKA rapidly phosphorylates Ira.

S2.1 Effect of Conditions (a), (b), and (c) on Equations (S3), (S7), and (S8)

Conditions (a), (b), and (c) simplify Equations (S3), (S7), and (S8), with results for Ma et al. **Cases 1–5** as follows.

Equation (S3)*:* In **Case 5**, both forms of both Pde1 and Pde2 are zero; therefore Equation (S3) can be rewritten as:

$$\frac{dz}{dt} \approx N_z y - D_z z, \tag{S12}$$

where

$$N_z = \frac{R_z C_z}{\Gamma_z + C_z}. \tag{S13}$$

When either Pde1 or Pde2 is nonzero:

$$\frac{R_z C_z}{\Gamma_z + C_z + \frac{\Gamma_z}{\Gamma_{p_1}} C_{p_1} + \frac{\Gamma_z}{\Gamma_{p_2}} C_{p_2}} \approx \frac{R_z C_z}{\frac{\Gamma_{p_1}}{\Gamma_z} C_{p_1} + \frac{\Gamma_{p_2}}{\Gamma_z} C_{p_2}} = \tilde{N}_z \tag{S14}$$

We can make this approximation since the last two terms in the denominator are large, by Condition (a). By Condition (c) we know R_z is also large so we can not approximate this term by zero; therefore in **Cases 1–4** Equation (S3) can be rewritten as:

$$\frac{dz}{dt} \approx \tilde{N}_z y - D_z z \tag{S15}$$

where $\tilde{N}_z << N_z$.

Equation (S7)*:* To apply Condition (a) examine the non-linear terms in the denominator of:

$$\frac{R_{p_1} y}{\frac{\Gamma_{p_1}}{C_{p_1}} + 1 + \frac{\Gamma_{p_1} C_{p_2}}{\Gamma_{p_2} C_{p_1}} + \frac{\Gamma_{p_1} C_c}{\Gamma_c C_{p_1}}}$$

Inequalities (S10) and (S11) allow us to neglect the last two terms in the denominator. Thus in **Cases 1, 3, and 4** Equation (S7) simplifies to:

$$\frac{dp_1}{dt} \approx N_1 y - D_{p_1} p_1, \tag{S16}$$

where

$$N_1 = \frac{R_{p_1}}{\frac{\Gamma_{p_1}}{C_{p_1}} + 1}.$$

In **Case 2** Pde1 is eliminated, so Equation (S7) is replaced by the trivial equation $p_1 \equiv 0$.

Equation (S8)*:* In **Case 2**, Pde1 has been eliminated ($p_1 \equiv 0$), and as above Inequalities (S10) and (S11) allow us to neglect the last term in the denominator of Equation (S8). Thus Equation (S8) simplifies to:

$$\frac{dp_2}{dt} \approx N_2 y - D_{p_2} p_2, \tag{S17}$$

where

$$N_2 = \frac{R_{p_2}}{\frac{\Gamma_{p_2}}{C_{p_2}} + 1}.$$

In all other Cases Equation (S8) is replaced by the trivial equation $p_2 \equiv 0$. In **Cases 1 and 4** this is because Pde1 is nonzero, so by Inequality (S10) the denominator is very large, and by Condition (b) the numerator is small, thus we can approximate the whole expression as zero. In **Cases 3 and 5** this is because Pde2 has been knocked out.

S2.2 Effect of Condition (b) on Equation (S17)

Condition (b) is imposed to fit **Cases 2 and 4** in the data reported by Ma et al. [35]. The slower activation of Pde2 is responsible for the larger transient response observed in these two cases (Figure 2). Applying this condition we can define a non-dimensional parameter

$$M = \frac{D_{p_2}}{D_{p_1}}, \tag{S18}$$

such that the value of M must be much less than one ($M \ll 1$). Changing the units of Pde2 if necessary, we can arrange that

$$N_2 = MN_1. \tag{S19}$$

Note that in every case at least one form of Pde has been eliminated. Since we have expressed the parameters in Equation (S17) as a constant multiple of the parameters in Equation (S16), we can express both as one equation:

$$\frac{dp}{dt} = M(N_1 y - D_1 p). \tag{S20}$$

In **Cases 1 and 3** the variable p represents Pde1 and the parameter M takes the value 1. In **Case 2** the variable p represents Pde2 and the parameter M is given by Equation (S18), which is much less than 1. In **Case 4** the variable p is again Pde1 but now parameter M is reduced to 0.2 since in this case the Pde1 mutant is slow activating. Of course in **Case 5**, the p-equation is not needed.

S2.3 Steady State Assumptions

The model may be further simplified by assuming that the following four reactions are fast and hence proceed to steady state.

1. Gpa2·GDP $\rightleftharpoons$ Gpa2·GTP (reaction 1 in Figure 1).

2. Activation/inactivation of Cdc25 (reaction 2 in Figure 1).

3. Activation/inactivation of adenylate cyclase (reaction 5 in Figure 1).

4. cAMP+[Bcy1::PKA] $\rightleftharpoons$ [cAMP::Bcy1] +PKA (reaction 9 in Figure 1).

Let us discuss the steady-state assumptions for each of these four reactions.

Gpa2·GDP $\rightleftharpoons$ Gpa2·GTP at steady state Gpa2·GTP is independent of all other variables, thus at steady state Gpa2·GTP is only a function of the glucose available to the cell:

$$g = \frac{P_g}{D_g}[\text{Glu}]. \tag{S21}$$

Activation/inactivation of Cdc25 at steady state Cdc25 is independent of all other variables, thus at steady state Cdc25 is only a function of the stress on the:

$$c = \frac{P_c}{D_c[\text{Stress}]} \tag{S22}$$

Thus stress inhibits the activity of Cdc25.

Activation/inactivation of adenylate cyclase at steady state

Applying this steady state assumption to (S5) we get that the steady state value of adenylate cyclase is:

$$a = \frac{P_a}{D_a} + \frac{\bar{P}_a P_g}{D_a D_g}[\text{Glu}]r \tag{S23}$$

cAMP+[Bcy1::PKA] $\rightleftharpoons$ [cAMP::Bcy1] +PKA at steady state In Equation (S9) we model this reaction as:

$$\frac{dy}{dt} = 2\left(K_b x^4 (C_y - \frac{y}{2}) - K_f y^3\right)$$

The reaction modeled here is: 4 cAMP bind to a single PKA unit, which is made up of two catalytic subunits (Tpk1, Tpk2, or Tpk3) and two regulatory subunits (Bcy1). cAMP binds to the regulatory subunits and releases the catalytic subunits creating the active form of PKA. We can simplify this reaction in two ways, first by assuming that the concentration of PKAdoes not approach its max ($C_y - \frac{y}{2} \approx C_y$), and second that we can approximate the 4 to 2 by a 2 to 1 reaction. This simplifies (S9) to:

$$\frac{dy}{dt} = 2(K_b x^2 - K_f y)$$

Although we know from Garmendia-Torres et al. [42] that this reaction (reaction 15 in Figure 1) is not faster than the other reactions in the system, we nevertheless make the simplest assumption consistent with the Ma et al. [35] data. Thus we assume that the activation/inactivation of PKA proceeds to steady state. We can then express the concentration of PKA (y) in terms of the concentration of cAMP (x):

$$y = \frac{K_b}{K_f} x^2$$

Of course models in which the reaction cAMP+[Bcy1::PKA] $\rightleftharpoons$ [cAMP::Bcy1] +PKA is not at steady state could fit the data of Ma et al. as well as our model or better. However, allowing this additional flexibility would lead to even more parameters needing to be chosen in the absence of sufficient data.

S3 Nondimensionalization

We seek to rescale our model in a way that is consistent in both the short term and long term. Conditions (a), (b) and (c) along with steady-state assumptions allow us to reduce Equations (S1)–(S9) to the following system:

$$\frac{dr}{dt} = \frac{R_r \frac{P_c}{D_c[Stress]}(C_r - r)}{\Gamma_r + C_r - r} - \frac{\bar{R}_r z r}{\Gamma_r + r} \quad \text{(S24a)}$$

$$\frac{dz}{dt} = N_z \frac{K_b}{K_f} x^2 - D_z z \quad \text{(S24b)}$$

$$\frac{dp}{dt} = M(N_1 \frac{K_b}{K_f} x^2 - D_1 p) \quad \text{(S24c)}$$

$$\frac{dx}{dt} = P_x \frac{P_a}{D_a} + \bar{P}_x \frac{\bar{P}_a P_g}{D_a D_g}[\text{Glu}]r - D_x x - \frac{R_x p x}{\Gamma_x + x} \quad \text{(S24d)}$$

In Equations (S24a)–(S24d) the variable p is the concentration of Pde1 in Cases 1, 3, and 4 (*wt*, *pde2*Δ, and *pde1*^{ala152}) and the concentration of Pde2 in Case 2; in Cases 1–4 parameter N_z is defined by (S13), and in Case 5 parameter N_z is defined by (S14); x is the concentration of cAMP; r is the concentration of Ras·GTP; and z is the concentration of Ira1 and Ira2; .

For convenance we rewrite these equations, the new parameters are now in bold.

$$\frac{dr}{dt} = \frac{\mathbf{R}_r(C_r - r)}{\Gamma_r + C_r - r} - \frac{\bar{R}_r z r}{\Gamma_r + r} \quad \text{(S25a)}$$

$$\frac{dz}{dt} = \mathbf{P}_z x^2 - D_z z \quad \text{(S25b)}$$

$$\frac{dp}{dt} = \mathbf{P}_p x^2 - D_p p \quad \text{(S25c)}$$

$$\frac{dx}{dt} = \mathbf{P}_x + \bar{\mathbf{P}}_x r - D_x x - \frac{R_x p x}{\Gamma_x + x} \quad \text{(S25d)}$$

To analyze (S25a)–(S25d), we introduce the following non-dimensional variables:

First re-scale such that $\bar{r} = \dfrac{r}{R}$, $\bar{z} = \dfrac{z}{Z}$, $\bar{p} = \dfrac{p}{P}$,$\bar{x} = \dfrac{x}{X}$, and $\bar{t} = \dfrac{t}{T}$ with the following parameters:

$$R = C_r \qquad Z = \frac{C_r D_p \bar{\mathbf{P}}_x \mathbf{P}_z}{R_x D_z \mathbf{P}_p} \qquad P = \frac{C_r \bar{\mathbf{P}}_x}{R_x}$$

$$X = \sqrt{\frac{C_r D_p \bar{\mathbf{P}}_x}{R_x \mathbf{P}_p}} \qquad T = \sqrt{\frac{D_p}{R_x \mathbf{P}_p \bar{\mathbf{P}}_x C_r}}$$

This scaling of variables yields the following non-dimensional parameters:

$$A = \frac{\sqrt{D_p}\mathbf{R}_r}{C_r^{3/2}\sqrt{R_x \mathbf{P}_p \bar{\mathbf{P}}_x}} \qquad B = \frac{D_p^{3/2}\bar{R}_r \mathbf{P}_z \sqrt{\bar{\mathbf{P}}_x}}{\sqrt{C_r} R_x^{3/2} D_z \mathbf{P}_p^{3/2}} \qquad \Gamma_1 = \frac{\Gamma_r}{C_r}$$

$$N = D_z \sqrt{\frac{D_p}{C_r R_x \mathbf{P}_p \bar{\mathbf{P}}_x}} \qquad M = D_p \sqrt{\frac{D_p}{C_r R_x \mathbf{P}_p \bar{\mathbf{P}}_x}} \qquad C = \frac{\mathbf{P}_x}{C_r \bar{\mathbf{P}}_x}$$

$$D_0 = D_x \sqrt{\frac{D_p}{C_r R_x \mathbf{P}_p \bar{\mathbf{P}}_x}} \qquad \Gamma = \frac{\Gamma_x \sqrt{R_x \mathbf{P}_p}}{\sqrt{C_r D_p \bar{\mathbf{P}}_x}}$$

We rewrite (S25a)–(S25d) in nondimensional form as:

$$\frac{dr}{dt} = \frac{A(1-r)}{\Gamma_1 + 1 - r} - \frac{Bzr}{\Gamma_1 + r} \tag{S26a}$$

$$\frac{dz}{dt} = N(x^2 - z) \tag{S26b}$$

$$\frac{dp}{dt} = M(x^2 - p) \tag{S26c}$$

$$\frac{dx}{dt} = C + r - D_0 x - \frac{Dpx}{\Gamma + x} \tag{S26d}$$

S4 Model Fitting

The parameters used to fit the dimensional model are listed in Table 1. In choosing dimensional parameters we used parameters given by Garmendia-Torres et al. [42] when available. The units have been converted from μM to fmol/(10^6 cells) using the approximation that the volume of a haploid cell is 42 fl while that of a diploid cell is 82 fl [63]. The haploid cell volume was used to fit the data reported by Ma et al., while the diploid cell volume was used to fit the experiemnts involving Σ1278b and S288c that we report here. Since the parameters of our model are non-dimensional this difference in cell volumes affected the scaling of the variables. When

fitting the Ma et al. data we scaled the concentration of cAMP by 24.95 fmol $\cdot 10^{-6}$ cells. For the diploid data we scaled by 48.71 fmol $\cdot 10^{-6}$ cells.

The parameters not chosen from Garmendia-Torres et al. are: D_1,P_x, D_x R_{x_1}, R_{x_2}, Γ_{x2} and M. Parameters D_1, P_x and R_{x_1} are fit to the Ma et al. wild type data, using a method of least squares. We used the Fortran subroutine lmdif.f as implemented in the MINPACK library [62]. This subroutine minimizes the sum of the squares of m nonlinear functions in n variables by a modification of the Levenberg-Marquardt algorithm. We created a subroutine which calculates the functions that are used by this algorithm. The Jacobian is then calculated by a forward-difference approximation.

Since Pde2 has higher affinity for cAMP than Pde1, the value of Γ_{x2} is half the value of Γ_{x1}. Paramters R_{x_2} and M where chosen to fit the Pde1 knockout case as described by Ma et al. again using a method of least squares. Finally the value of D_x was chosen to fit the steady-state value of cAMP in the double Pde knockout case.

[Table 1 about here.]

S5 Long-Term Dynamics

We explore the possible long-term dynamics of cAMP by examining the full four by four system:

$$\frac{dr}{dt} = \frac{A(1-r)}{\Gamma_1 + 1 - r} - \frac{Bzr}{\Gamma_1 + r} \tag{S27a}$$

$$\frac{dz}{dt} = N(x^2 - z) \tag{S27b}$$

$$\frac{dp}{dt} = M(x^2 - p) \tag{S27c}$$

$$\frac{dx}{dt} = C + r - D_0 x - \frac{Dpx}{\Gamma + x} \tag{S27d}$$

Claim 1. *Equations* (S27a)–(S27d) *have a unique positive equilibrium point for* $0 < r < 1$.

Proof. To prove this, we will set Equations (S27a)–(S27d) equal to zero and show that in the range $0 < r < 1$ there exists a unique positive solution (r_{ss}, z_{ss}, p_{ss}, x_{ss}). By setting Equations (S27b) and (S27c) equal to zero we can express variables p and z in terms of the x:

$$z = p = x^2.$$

Substituting $p = x^2$ into Equation (S27d) and setting this equation equal to zero, we can solve for variable r in terms of x:

$$r = \frac{1}{G}\left(D_0 x + \frac{Dx^3}{\Gamma + x} - C\right). \tag{S28}$$

Substituting this expression for r in terms of x, and $z = x^2$ into Equation (S27a) and setting this equation equal to zero we reduce the four-by-four system to a single equation that we can solve for in terms of x. For convenience of notation define $f(x)$ as given by Equation (S28), then Equation (S27a) set equal to zero becomes:

$$0 = \frac{A(1 - f(x))}{\Gamma_1 + 1 - f(x)} - \frac{Bx^2 f(x)}{\Gamma_1 + f(x)}. \tag{S29}$$

Then, we need to show that there exists a unique solution to Equation (S29). To do this we first examine the behavior of $f(x)$. Observe that $f(x)$, given by Equation (S28), is a continuous, monotonically increasing function for x greater than zero. Since

$$f(0) = -\frac{C}{G} \qquad \text{and} \qquad f(x) \to \infty \text{ as } x \to \infty,$$

there exists unique positive real points x_0 and x_1 such that $f(x_0) = 0$ and $f(x_1) = 1$.

Then to solve Equation (S29) define

$$h(x) = \frac{A(1 - f(x))}{\Gamma_1 + 1 - f(x)} \qquad \text{and} \qquad g(x) = \frac{Bx^2 f(x)}{\Gamma_1 + f(x)}$$

and show that there is a unique solution to

$$h(x) = g(x) \qquad \text{for } x_0 < x < x_1.$$

Observe that

$$h(x_0) = \frac{A}{\Gamma_1 + 1} > 0, \qquad h(x_1) = 0,$$

and

$$h'(x) = \frac{-A\Gamma_1 f'(x)}{(\Gamma_1 + 1 - f(x))^2} < 0 \qquad \text{for } x_0 < x < x_1.$$

Thus, $h(x)$ is a continuous, monotonically decreasing function for $x_0 < x < x_1$.

Observe that

$$g(x_0) = 0, \qquad g(x_1) > 0,$$

and

$$g'(x) = \frac{\Gamma_1(2Bxf(x) + Bx^2 f'(x)) + 2Bx(f(x))^2}{(\Gamma_1 + f(x))^2} > 0 \qquad \text{for } x_0 < x < x_1.$$

Thus, $g(x)$ is a continuous, monotonically increasing function for $x_0 < x < x_1$.

Therefore, there exists a unique solution x_{ss} to $h(x) = g(x)$ such that $x_0 < x_{ss} < x_1$. Then the unique positive equilibrium point of Equations (S27a)–(S27d), implicitly defined in terms of x_{ss}, is $(f(x_{ss}), x_{ss}^2, x_{ss}^2, x_{ss})$. Since $0 < f(x_{ss}) < 1$ the equilibrium value of variable r is restricted to the range $0 < r < 1$.

□

Then using this outline, we can numerically find our equilibrium point for any parameter choice.

Next we want to look at the stability of this equilibrium point. To do so we examine the Jacobian Matrix at the equilibrium point:

$$\mathbf{J} = \begin{pmatrix} -\frac{\Gamma_1 A}{(\Gamma_1+1-r_{ss})^2} - \frac{\Gamma_1 B x_{ss}^2}{(\Gamma_1+r_{ss})^2} & -\frac{Br_{ss}}{\Gamma_1+r_{ss}} & 0 & 0 \\ 0 & -N & 0 & 2Nx_{ss} \\ 0 & 0 & -M & 2Mx_{ss} \\ 1 & 0 & -\frac{Dx_{ss}}{\Gamma+x_{ss}} & -D_0 - \frac{\Gamma D x_{ss}^2}{(\Gamma+x_{ss})^2} \end{pmatrix}. \tag{S30}$$

Claim 2. *In the special case of $M = N$ Equations* (S27a)–(S27d) *pass through a Hopf Bifurcation when*

$$\frac{Br_{ss}}{\Gamma_1 + r_{ss}} = \frac{(a^2 + aTr + Det)Tr}{2x_{ss}M} \tag{S31}$$

where r_{ss} and x_{ss} are the steady state values of Ras·GTP and cAMP,

$$a = \frac{\Gamma_1 A}{(\Gamma_1 + 1 - r_{ss})^2} + \frac{\Gamma_1 B x_{ss}^2}{(\Gamma_1 + r_{ss})^2} \qquad Tr = M + D_0 + \frac{\Gamma D x_{ss}^2}{(\Gamma + x_{ss})^2}$$

$$Det = M(D_0 + \frac{\Gamma D x_{ss}^2}{(\Gamma + x_{ss})^2} + 2\frac{D x_{ss}^2}{\Gamma + x_{ss}})$$

that is Tr is the absolute value of the Trace of the two by two matrix determined by Pde and cAMP, and Det is the determinant of the same matrix.

Proof. We will just outline the proof here. Let:

$$a = \frac{\Gamma_1 A}{(\Gamma_1 + 1 - r_{ss})^2} + \frac{\Gamma_1 B x_{ss}^2}{(\Gamma_1 + r_{ss})^2} \qquad b = \frac{Br_{ss}}{\Gamma_1 + r_{ss}}$$

$$d_1 = \frac{Dx_{ss}}{\Gamma + x_{ss}} \qquad d_2 = D_0 + \frac{\Gamma D x_{ss}^2}{(\Gamma + x_{ss})^2}$$

then we rewrite the Jacobian Matrix as:

$$\mathbf{J} = \begin{pmatrix} -a & -b & 0 & 0 \\ 0 & -M & 0 & 2Mx_{ss} \\ 0 & 0 & -M & 2Mx_{ss} \\ 1 & 0 & -d_1 & -d_2 \end{pmatrix}.$$

The characteristic polynomial of this matrix has the following form:

$$(\lambda + M)\left((\lambda + a)(\lambda^2 + (M + d_2)\lambda + M(d_2 + 2x_{ss}d_1) + 2bx_{ss}M)\right) = 0$$

Then the first eigenvalue is $\lambda = -M$. We then used Mathematica to solve the remaining cubic. We find that the eigenvalues pass through a Hopf Bifurcation when:

$$b = \frac{Tr(a^2 + aTr + det)}{2x_{ss}M}$$

by solving for when the eigenvalues are complex with zero real part. □

S6 Short-Term Dynamics

In either extream case of $r_{ss} \approx 1$ (Ras almost completely active -in GTP form) or $r_{ss} \approx 0$ (Ras almost completely inactive -in GDP form) we can assume that variable r is at steady state and reduce the four–by–four system to the following two–by–two system:

$$\frac{dp}{dt} = M(x^2 - p) \tag{S32a}$$
$$\frac{dx}{dt} = C_0 - D_0 x - \frac{Dpx}{\Gamma + x}. \tag{S32b}$$

where $C_0 = C + G$ when $r_{ss} \approx 1$ (Ras is almost completely active) and $C_0 = C$ when $r_{ss} \approx 0$ (Ras is almost completely inactive). Since these two cases vary only by the value of parameter C_0 we analyze the behavior of both cases simultaneously.

Claim 1. *Solutions of Equations* (S32a) *and* (S32b) *exhibit an oscillatory approach to equilibrium if and only if*

$$\left(M - D_0 - \frac{\Gamma D x_{ss}^2}{(\Gamma + x_{ss})^2}\right)^2 < 8M\frac{Dx_{ss}^2}{\Gamma + x_{ss}}. \tag{S33}$$

where x_{ss} is the equilibrium solution of (S32a, S32b); i.e.,

$$C_0 - D_0 x_{ss} - \frac{Dx_{ss}^3}{\Gamma + x_{ss}} = 0. \tag{S34}$$

Proof. To determine the parameter range for which (S32a) and (S32b) exhibit decaying oscillations, we examine the Jacobian matrix (S35) determined by (S32a) and (S32b) evaluated at the equilibrium point:

$$\mathbf{J} = \begin{pmatrix} -M & 2Mx_{ss} \\ -\frac{Dx_{ss}}{\Gamma + x_{ss}} & -D_0 - \frac{\Gamma D x_{ss}^2}{(\Gamma + x_{ss})^2} \end{pmatrix}. \tag{S35}$$

When (S35) has complex eigenvalues with negative real part, then the system will exhibit decaying oscillations as it approaches the equilibrium. The trace and determinant of our Jacobian matrix are:

$$\text{Trace}(J) = -M - D_0 - \frac{\Gamma D x_{ss}^2}{(\Gamma + x_{ss})^2} \tag{S36}$$

$$\det(J) = M(D_0 + \frac{\Gamma D x_{ss}^2}{(\Gamma + x_{ss})^2} + \frac{2Dx_{ss}^2}{\Gamma + x_{ss}}) \tag{S37}$$

A two-by-two matrix has complex eigenvalues when

$$\text{Trace}(J)^2 < 4\det(J).$$

Thus using (S36) and (S37), we can state that (S32a) and (S32b) exhibit decaying oscillations when:

$$\left(M + D_0 + \frac{\Gamma D x_{ss}^2}{(\Gamma + x_{ss})^2}\right)^2 < 4M\left(D_0 + \frac{\Gamma D x_{ss}^2}{(\Gamma + x_{ss})^2} + 2\frac{Dx_{ss}^2}{\Gamma + x_{ss}}\right). \tag{S38}$$

We can simplify (S38) by subtracting $4M(D_0 + \frac{\Gamma D x_{ss}^2}{(\Gamma + x_{ss})^2})$ from both sides. The left hand side remains a complete square, and we can rewrite (S38) as

$$\left(M - D_0 - \frac{\Gamma D x_{ss}^2}{(\Gamma + x_{ss})^2}\right)^2 < 8M\frac{Dx_{ss}^2}{\Gamma + x_{ss}}.$$

□

Tables

Parameter	Value	Unit	Description
R_r	240	$(\text{min})^{-1}$	activation rate of Ras·GTP due to Cdc25
$\bar{R}_r$	120	$(\text{min})^{-1}$	inactivation rate of Ras·GTP due to Ira
C_r	1050	fmol/(10^6 cells)	total concentration of Ras
Γ_r	.42	fmol/(10^6 cells)	affinity of Cdc25 and Ira for Ras
P_c	168	fmol/(10^6 cells)$(\text{min})^{-1}$	activation rate of Cdc25
D_c	1	$(\text{min})^{-1}$	basal inactivation rate of Cdc25
P_z	.7	$(\text{min})^{-1}$	activation rate of Ira due to PKA
D_z	.86	$(\text{min})^{-1}$	inactivation rate of Ira
P_a	1.25	fmol/(10^6 cells) $(\text{min})^{-1}$	basal activation rate of AC
$\bar{P}_a$	.027	$(\text{min})^{-1}$	enhanced activation rate of AC
D_a	1	$(\text{min})^{-1}$	inactivation rate of AC
R_{p_1}	2	$(\text{min})^{-1}$	activation rate of Pde1 due to PKA
$\mathbf{D_{P_1}}$	.25	$(\text{min})^{-1}$	inactivation rate of Pde1
K_b	.007	(min fmol/(10^6 cells)$)^{-1}$	rate at which cAMP binds to PKA
K_f	3	$(\text{min})^{-1}$	rate at which cAMP is released from PKA
$\mathbf{M}$	.047	–	ratio between the inactivation rates of Pde1 and Pde2
$\mathbf{P_x}$	23.3	$(\text{min})^{-1}$	production rate of cAMP due to AC
$\mathbf{D_x}$	.34	$(\text{min})^{-1}$	basal decay rate of cAMP
$\mathbf{R_{x_1}}$	56.8	$(\text{min})^{-1}$	decay rate of cAMP due to active Pde1
Γ_{x_1}	840	fmol/(10^6 cells)	affinity of Pde1 for cAMP
$\mathbf{R_{x_2}}$	14.7	$(\text{min})^{-1}$	decay rate of cAMP due to active Pde2
$\mathbf{\Gamma_{x_2}}$	420	fmol/(10^6 cells)	affinity of Pde2 for cAMP

Table 1: Parameter values used to fit (S24a)–(S24b) to the data from Ma et al. [35]. Parameter in bold are parameters that are fit to the Ma et al. datat using a method of least-squares. Parameters P_x, D_1 and R_{x_1} were fit the the wild-type, parameters R_{x_2} and M were fit to the Pde1 knockout case, and D_x was determined to fit the double Pde knockout case. The reminin parameters were taken from Garmendia-Torres et al.
[42]
